# Differential alternative splicing in skeletal muscle after 14 days immobilization-induced atrophy in young men

**DOI:** 10.1007/s00424-026-03221-9

**Published:** 2026-10-12

**Authors:** Aliah Y. Aziz, Jamie-Lee M. Thompson, Thomas M. Doering, Paul J. Dunn, Vernon G. Coffey

**Affiliations:** 1 Bond Institute of Health and Sport and Faculty of Health Sciences and Medicine, Bond University, 2 Promethean Way, Robina, Gold Coast, Queensland 4226 Australia; 2 Victor Chang Cardiac Research Institute, Darlinghurst, Sydney, Australia; 3 St Vincent’s Clinical School, Faculty of Medicine, UNSW, Sydney, Australia; 4 School of Health, Medical and Applied Sciences, Central Queensland University, Queensland Rockhampton, Australia

**Keywords:** Muscle wasting, Unloading, Disuse, Catabolic, Genes

## Abstract

Skeletal muscle disuse during injury decreases muscle mass and function. Previous studies have determined differential gene expression associated with atrophy, but changes in alternative splicing (AS) in response to unloading is poorly defined. This study aimed to identify global changes in differential alternative splicing (DAS) in skeletal muscle following unloading. Twenty-one healthy males (26.0 ± 5.7 years; 81.2 ± 11.6 kg) underwent 14 days unilateral knee-brace immobilization. Participants underwent strength and muscle mass assessment before and after the immobilization and vastus lateralis muscle biopsies were taken pre-immobilization, and on days 3 and 14. RNA-sequencing was performed, and DAS events were identified using the rMATS package. Leg strength decreased − 21.5 kg (*P* < 0.0001) while mid-thigh circumference was reduced ~ 2% (*P* = 0.016). A total of 157,464 and 159,395 splicing events affecting 15,219 and 15,374 genes identified on day 3 and 14, respectively. Most DAS events occurred in coding sequence regions and were primarily due to exon skipping. A higher number of DAS events were observed by day 14 than day 3, indicating time-dependent regulation. Gene Ontology analysis of DAS at day 14 revealed 37 enriched GO biological processes related to MAPK signalling, mitochondrial function, and organelle organization. Unloading-induced atrophy was associated with widespread changes in AS, particularly in genes linked to muscle maintenance and protein synthesis. AS appears to promote down-regulation of target genes and subsequent differentially enriched biological pathways associated with the functional outcomes of decreased muscle mass and strength. These findings indicate changes in alternative splicing is a regulatory mechanism in muscle wasting during immobilization.

**Supplementary Information:**

The online version contains supplementary material available at https://doi.org/10.1007/s00424-026-03221-9.

## Introduction

Previous studies have investigated skeletal muscle atrophy through large-scale differential gene expression (DGE) studies, but the regulatory processes involved are complex and remain poorly understood. Alternative splicing (AS), an important mechanism for eukaryotic gene regulation, has a putative role as a major contributor to regulation of the transcriptome [1]. As such, splice-altering variants could be an important factor in regulation of skeletal muscle atrophy. Alternative splicing events are classified as skipped exons (SE), alternative 5’ splice sites (A5SS), alternative 3’ splice sites (A3SS), mutually exclusive exons (MEX), and retained introns (RI) [2]. Of these five mechanisms, SE is the most common, accounting for ~ 40% of the global splicing events in humans [2]. The SE event is characterised by the inclusion or exclusion of a skipped alternative exon from the mature mRNA [3]; A5SS/A3SS mechanisms are apparent when there are multiple splice sites at either the donor 5’ site or acceptor 3’ site, or site of the exon that can be used to create a different protein isoform [4]; MEX splicing occurs in a coordinated manner where a transcript retains only one of two alternative exons, or one of two exon groups, into the mature mRNA; RI is when the intron excision is delayed or inhibited, and therefore not removed from the mature mRNA [4].

Tissue-wide studies of alternative splicing show skeletal muscle has one of the highest numbers of tissue-specific splicing events [5]. Given the extent of splicing in skeletal muscle, it has been proposed that variants that disrupt alternate exon selection may contribute to muscle degeneration during unloading [2]. Moreover, two previous animal studies have characterised differential alternative splicing (DAS) events associated with muscle unloading, but there is a paucity of data to support similar responses in humans [6, 7]. Accordingly, whether differential alternative splicing occurs in response to muscle unloading in humans has not been established. The aim of the present study was to determine changes in AS events associated with 14 days of knee brace immobilization in healthy, physically active young men. The hypothesis was that the reduced muscle contractile activity due to limb immobilization would generate a differential alternative splicing profile associated with the loss of skeletal muscle mass.

## Method

### Ethics approval

The study conformed to the standards set by the Declaration of Helsinki. The procedures have been approved by the Bond University Human Research Ethics Committee (HREC#0000015478). The study was registered as a clinical trial on 10 October 2016 with the Australian New Zealand Clinical Trials Registry (ACTRN12616001399482).

### Consent

Written informed consent was obtained from all individual participants included in the study prior to their participation.

### Overview

The experimental trial period was preceded by a standardised exercise program to equilibrate the relative exercise history and muscle adaptive state of participants prior to limb immobilization [8]. In addition, one week before the experimental period participants began a three-week dietary control to achieve matched relative energy intake equivalent to estimated daily energy expenditure during immobilization. These design features ensured the study is highly-controlled for habitual diet and physical activity. Detailed methods of the experimental design have been previously described elsewhere [8].

### Participants

Twenty-one young, apparently healthy men volunteered to take part in this study. Briefly, participants were 20–40 year (mean 26.0 ± 5.7 year) with mean body mass 81.0 ± 11.6 kg and height 179.5 ± 6.8 cm. A commercial meal delivery system (direct to home) was contracted to provide primary meals and snacks for participants during the dietary control period (24% protein; Light n’ Easy, Brisbane, Australia). Participants were not permitted to consume any additional food or calorie-containing fluid outside of their prescribed diet. Food provided aimed to achieve energy balance based on the Schofield equation and a physical activity level (PAL) of 1.5 [9].

### Skeletal muscle strength and size

Quadriceps circumference was recorded manually at the mid-thigh anatomical site by tape measure. The same site was used to determine vastus muscle group cross-sectional area (CSA) via magnetic resonance imaging (MRI; 3T Magnetom Skyra, Siemens Healthineers, Victoria, Australia). Five × 5 mm slices were imaged from proximal to distal with 2 mm gaps (field of view = 380 mm; resolution = 336 × 448) for which the middle image was subsequently analysed by muscle tracing using OsiriX Lite 8.0 software [10] (Pixmeo, Bernex, Switzerland). Limb muscle strength was determined using the three repetition maximum method on a 45° leg press machine (Hammer Strength, Rosemont, Illinois).

### Limb immobilization and muscle sampling

Participant’s left knee joint was immobilized using a telescoping adjustable knee brace which were individually fitted at 60˚ flexion and secured with a single-use fastener (X-ACT DonJoy Orthopedics, Texas, USA). Forearm crutches were also provided to avoid bearing any weight on the immobilized limb. Muscle biopsies were taken from the left leg vastus lateralis immediately prior to immobilization at day 0, and at day 3 and day 14 post immobilization. The brace was not removed at any point during the immobilization except for the biopsy on day 3 and each sample was obtained from a different site/incision from distal to proximal. Muscle samples were obtained in the morning after overnight fast at the same relative time of day.

### RNA extraction and sequencing

Total RNA was isolated from 63 muscle biopsies (21 individuals, 3 time-points) using the AllPrep DNA/RNA/miRNA Universal kit (QIAGEN) according to manufacturer’s instructions and stored at -80 °C [11]. RNA quantity and quality was assessed via UV spectrophotometry (NanoDrop) and fluorometry (Qubit 3), A260/A230 and A260/A280 ratios of > 1.8 and > 2.0, respectively, indicated high-quality RNA. RNA integrity was assessed using RNA 6000 Nano kit on a 2100 Bioanalyser (Agilent). A RIN of 7.0 was considered acceptable for RNA sequencing with all RNA samples above this threshold. Total RNA was stored at -80 °C prior to further analysis.

RNA sequencing was performed by the Australian Translational Genomics Centre located at Queensland University of Technology/Translational Research Institute. Briefly, 600 ng of each RNA sample was used for library construction using the TruSeq Stranded Total RNA Library kit with Ribo-Zero Gold rRNA depletion (Illumina) as per manufacturer’s instructions. Adapter-ligated fragments were amplified by PCR for 11 cycles and the quality and size of the final library preparations analysed on a TapeStation (Agilent). Indexed samples were pooled and then sequenced on a NextSeq 500 system (Illumina) across 8 flow cells, generating approximately 62 million paired-end 2 × 75 bp reads per sample. Following sequencing the data was demultiplexed and the resulting FASTQ files used for bioinformatics analysis.

### Bioinformatic analysis

Filtering and mapping of sequencing data.

Quality control of the raw FASTQ files was performed using FASTQC software package version 0.12.1 [12]. No additional trimming was necessary after QC was assessed. Transcript quantification and mapping to the human reference transcriptome (cDNA and ncRNA) using Ensembl GRCh38 release 109 (www.ensembl.org) was performed with STAR software package version 2.7.1 [13] using the default parameters.

#### Identification of alternative splicing events

The alternative splicing events were detected and classified as percent spliced by the rMATS [1] software using junction. Alternative splicing events were divided into the following 5 categories using rMATS: SE, A3SS, A5SS, MEX, RI. Junction counts were used for each AS event detection where the value of the inclusion level (IncLevel) was used to define the degree of alternative splicing. To be included as part of the analysis, the ΔIncLevel between the experimental and control groups at each timepoint was > 0.01, with a false discovery rate (FDR) ≤ 0.05 [7]. The total splicing activation and inhibition spliced transcripts were also measured with splicing activation defined as ΔIncLevel > 0 and splicing inhibition defined as ΔIncLevel difference < 0. This analysis was conducted to allow for direct comparison with work by Sun and co-workers employing an animal atrophy model of hindlimb unloading [7]. Additional analyses to determine total DAS events at higher thresholds of ΔIncLevel > 0.05, and ΔIncLevel > 0.10 is included as supplementary material (supplementary file S1).

Differential gene expression analysis was conducted on all genes to determine directionality of expression with AS events. Genes with low coverage depth based on a median count per million of < 0.5 across all 63 samples were excluded from the analysis. Trimmed mean of M values (TMM) normalisation was used to correct for composition bias and library size before voom transformation and descriptive statistics. Differential expression analysis using linear modelling and empirical Bayes methods was carried out in LIMMA version 3.46.0 [14]. Paired sample comparisons were employed using participant ID as a blocking factor in the design matrix, to compare immobilised (day 3 or day 14) and baseline (day 0) states within participants. The linear model also incorporated RIN as a covariate. A false discovery rate (Benjamini-Hochberg) was applied to correct for multiple testing, with statistical significance accepted at FDR < 0.05. Principal Component Analysis (PCA) was performed on the AS datasets generated from all three days and between day 0 to day 3 and day 0 to day 14.

### Gene ontology (GO) analysis

The CLUSTERPROFILER tool in R was used to analyse the GO enrichment function of overlapping alternative splicing genes to understand the function of these genes. Briefly, using the Gene Set Enrichment Analysis (GSEA) algorithm, all analysed genes were ranked (from the most upregulated to the most downregulated) in order of evidence using the signed moderated t-statistic from LIMMA [14]. This identified pre-specified sets of differentially alternative spliced genes that were enriched at the top (i.e., upregulated) or at the bottom (i.e., downregulated) of this ranked list. Over-representation analysis of these gene lists using the CLUSTERPROFILER package version 4.12.6 [15] was conducted to reveal GO analysis pathways affected by significant gene expression changes as well as AS changes.

### Statistical analysis

Data for skeletal muscle strength and size before and after the 14 day immobilization period were analysed via paired t-test using GraphPad Prism (v10.1.2).

## Results

### Skeletal muscle strength and size

The 14 day limb immobilization-induced atrophy resulted in a substantial loss of skeletal muscle strength, external circumference of the thigh, and the anatomical cross-sectional area of the primary muscles of the quadriceps femoris (Table 1). Specifically, there was a decrease in mean three repetition maximum leg press muscle strength of -21.5 kg (*P* < 0.0001), mid-thigh circumference − 1.09 cm (*P* = 0.016) and muscle cross sectional area − 6.96 cm^2^ (*P* < 0.0001).

**Table 1:** Skeletal muscle strength, thigh circumference and muscle cross sectional area (CSA) of healthy, young men (*n* = 21) before (pre) and after (post) 14 days knee brace limb immobilization (IMM). Data are mean (±) standard deviation

	Pre-IMM		Post-IMM		∆%
Leg press strength (kg)	130.4	± 26.0	108.9**	± 25.6	-16.4
Mid-thigh circumference (cm)	54.49	± 4.90	53.39*	± 4.84	-2.01
Vastus muscle group CSA (cm^2^)	77.63	± 10.68	70.67**	± 10.03	-9.0

Post-IMM significantly different from Pre-IMM *P<0.05, **P<0.0001. Leg strength determined by three repetition maximum method; vastus muscle group includes vastus lateralis, intermedius and medialis oblique

### RNA sequencing

**Fig. 1:**
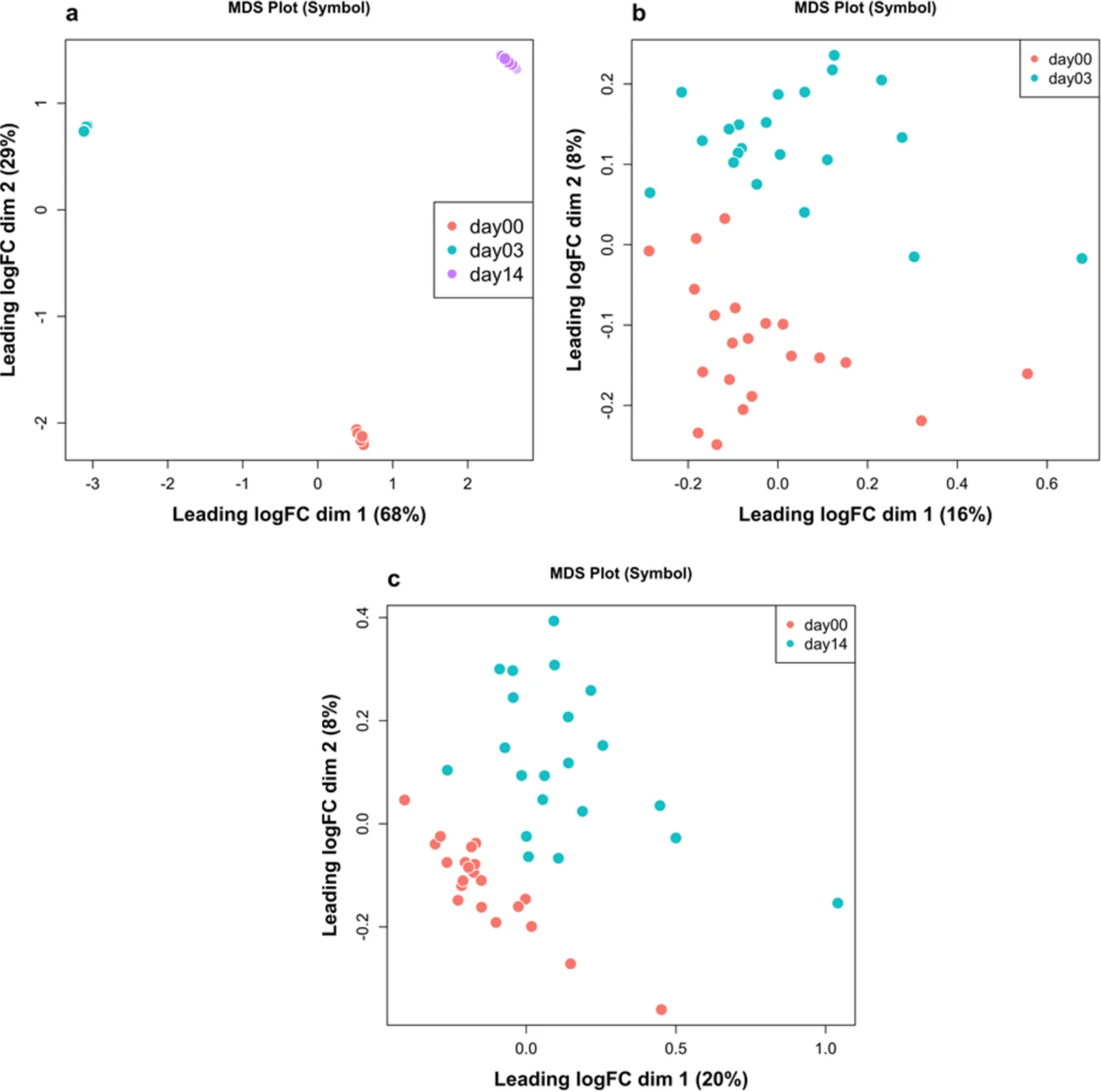
Principal component analysis (PCA) showing the two major components (PC1 vs. PC2) following the 14 days of limb immobilization. RNA-sequencing analysis of the total AS events from the *vastus lateralis* muscle is shown in response to immobilization at (**a**) three different timepoints (before and after day 3 and day 14 of immobilization), (**b**) between baseline day 0 and day 3, and (**c**) between baseline day 0 and day 14

RNA sequencing generated an average of 62.6 million mapped reads (range = 55.2–76.5 million). FASTQC analysis showed all 63 samples were successfully sequenced with per-base sequence quality cores consistently exceeding 30. Following normalisation and adjustments of the raw data, there is a clear separation at the three different timepoints (day 0, day 3 and day 14). Principal component analysis (PCA) illustrated the variance in gene expression between the muscle sampling timepoints (Fig. 1). Specifically, PC2 showed the clear separation between the different timepoints, indicating that the duration of immobilization of the limb has a significant effect on gene variability. Notably, there was more similarity between day 3 and day 14 than there was with the baseline control timepoint.

### Alternative splicing

**Fig. 2:**
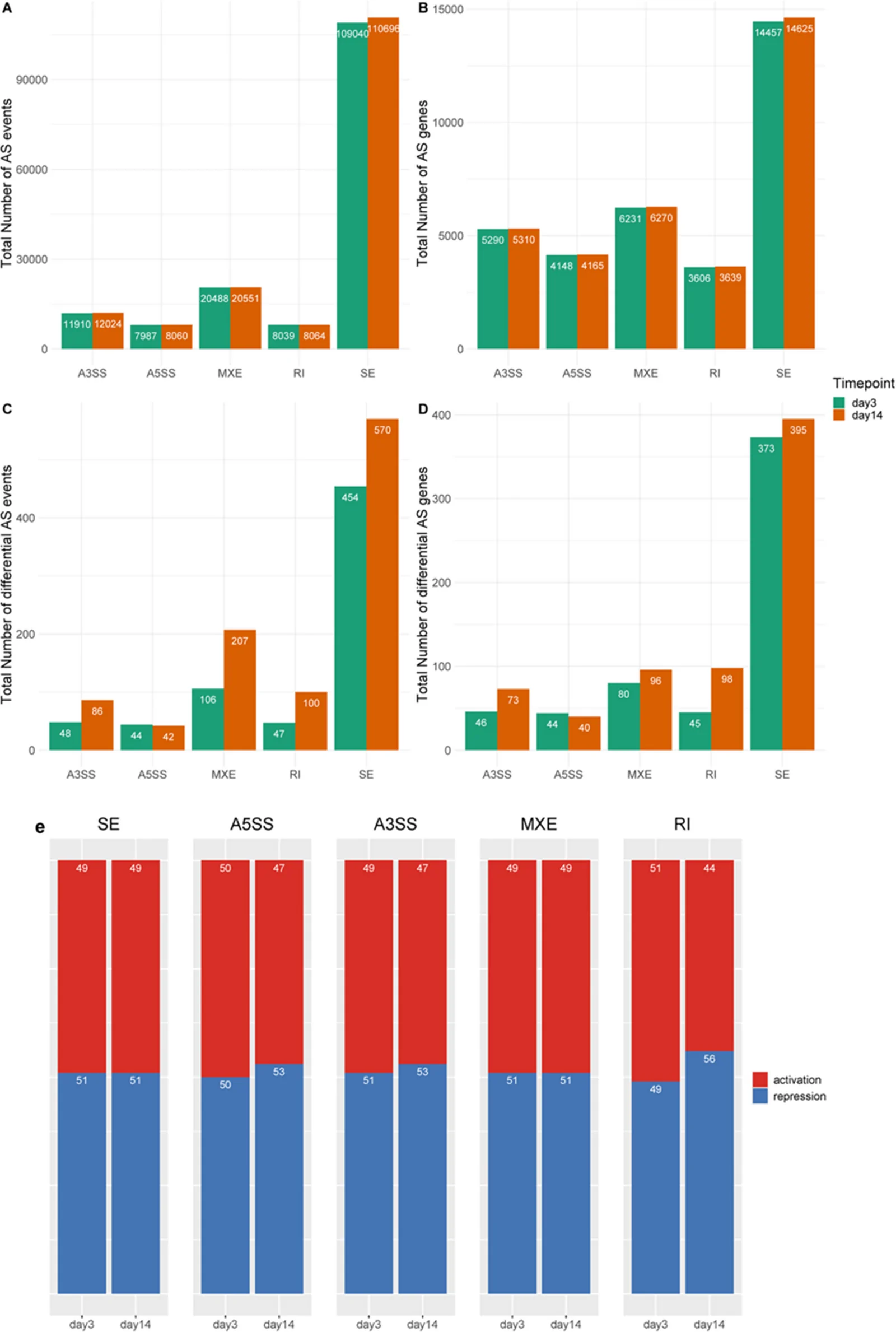
Differential alternative splicing events in skeletal muscle during 14 days knee brace immobilization in healthy young men (n = 21). Muscle biopsies were obtained from vastus lateralis and samples from day 0 were used as the baseline comparison for day 3 and day 14. (**a**) Total number of splicing events; (**b**) total number of genes corresponding to the total AS events; (**c**) number of differential AS events; (**d**) number of differential alternative splicing genes for each AS event; (**e**) number and proportion (percent) of the alternative splicing forms that activate or inhibit alternative splicing events. Blue box represents splicing repression, and red box represents splicing activation. SE, skipped exon; A5SS, alternative 5’ splicing site; A3SS, alternative 3’ splicing site; MXE, mutually exclusive exon; RI, retained introns

In response to limb immobilization, a total of 157,464 (day 3) and 159,395 (day 14) alternative splicing events were identified, which corresponded to a total of 15,219 and 15,374 genes at day 3 and day 14, respectively. Compared to baseline, there were 699 and 1005 differential alternative splicing events at day 3 and 14 that were identified, which matched to 533 and 628 genes, respectively. Of these, SE was the main alternative splicing mode and accounted for > 70%, regardless of the event or gene (Fig. 2a, b). While the total number of events and genes were comparable, the differences between day 3 and day 14 at an individual gene level was more apparent. Specifically, at day 14 there was significantly more differential AS events and AS genes compared to day 3 (Fig. 2c, d). The difference between the differential splicing events at both timepoints were analysed. The results showed that for the alternative splicing events in the MEX and SE modes, the proportions of activation and inhibition were not significantly different. Compared with baseline (day 0), the inhibited/repressed alternative splicing events accounted for a relatively larger number in the later stage of atrophy for the RI model, reaching 56% at day 14 compared to the 49% at day 3 (Fig. 2e).

### Gene ontology analysis

**Fig. 3:**
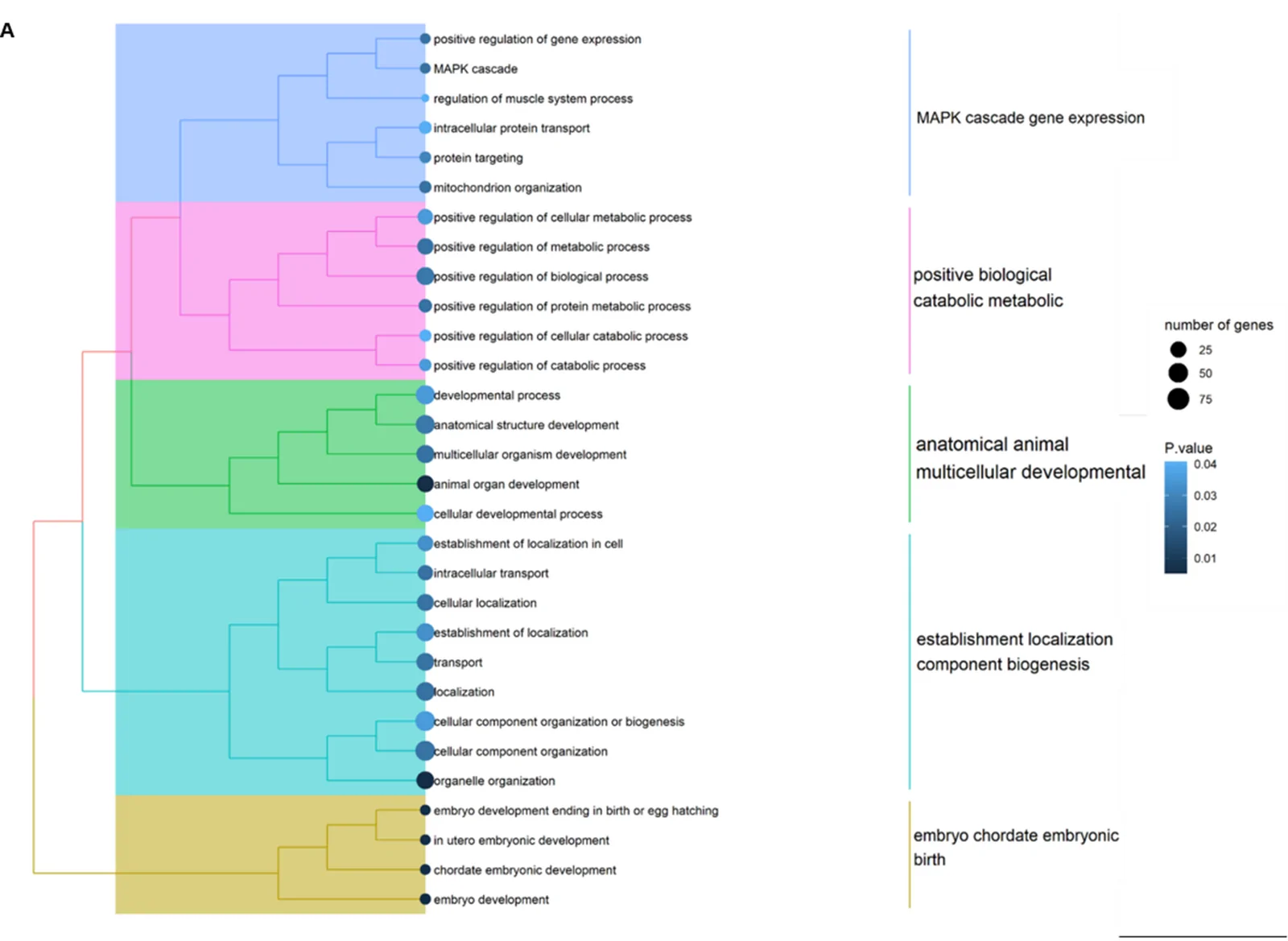

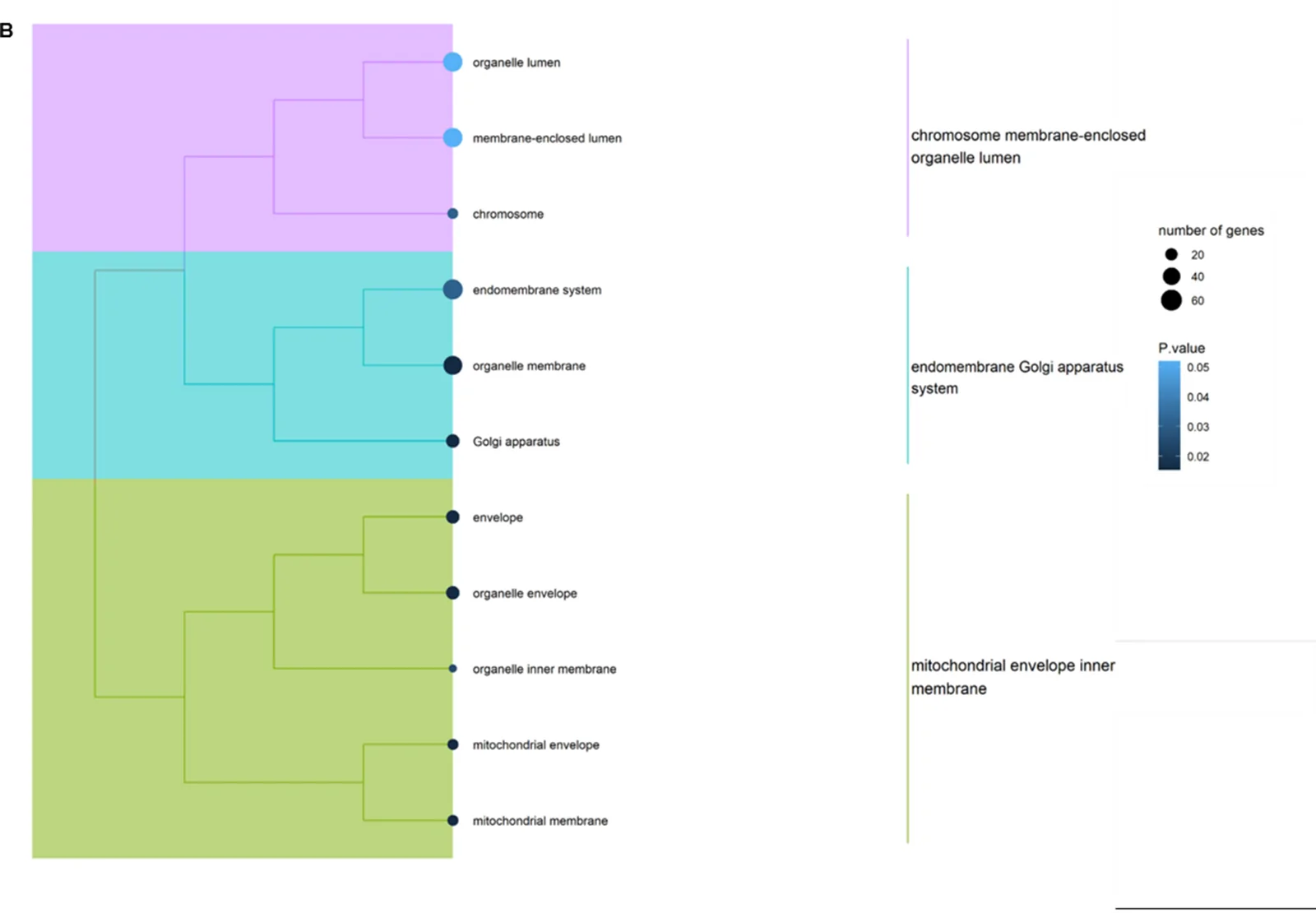
Gene Ontology (GO) analysis of the downregulated differential alternative splicing genes in all five splicing modes (SE, MXE, RI, A5SS, A3SS) at day 14 of knee brace immobilization from vastus lateralis muscle biopsy samples in young healthy men (*n* = 21). The top 30 most significant terms for biological process (BP) were (**a**) classified into five groups including MAPK cascade gene expression (blue), positive biological catabolic metabolic process (pink), anatomical animal multicellular developmental (green), establishment localization component biogenesis (teal), and embryo chordate embryonic birth (yellow). The significant cellular components (CC) shown (**b**) are categorised into three clusters: chromosome organelles (purple), endomembrane system (teal), and mitochondrial envelope (green). The dot plots show the significance of the p-value (*P* < 0.05 − 0.01) on a blue colour gradient scale and the number of genes in each pathway represented by the size of circle

As muscle atrophy is a gradual process and cells continue to maintain basal level of viability and function, the differentially alternative spliced genes at day 14 were further characterised through gene ontology (GO) analysis. There was no significant GO terms found at day 3 nor were there significant GO terms when splicing mechanisms (e.g., skipped exons or retained introns) were analysed independently, therefore, only day 14 results encompassing the collective of differentially alternative spliced genes are presented. The top 30 most significant GO terms for biological process (BP) and top 20 most significant GO terms for cellular components at day 14 were identified. The negatively enriched GO BPs were classified into five groups including mitogen-activated protein kinase (MAPK) cascade gene expression (blue), positive catabolic and metabolic process (pink), cellular development (green), localization of components (teal), embryo development (yellow) (Fig. 3a). The most significant pathways identified were all within the embryo development group except for animal organ development and organelle organization. All the catabolic pathways involved were positive regulation of either metabolic, biological or catabolic processes. As they were all downregulated, alternative splicing as a mechanism was involved in attenuation of these pathways.

The most significant GO cell components were the mitochondrial membrane, Golgi apparatus, organelle membrane, mitochondrial membrane and organelle envelope and they were all downregulated at day 14 compared to baseline (Fig. 3b). The most significant cell components also correlate to the high number of developmental GO BP pathways due to the contribution of mitochondria and energy-related pathways as well as the localization and transport pathways related to the Golgi apparatus and organelle membrane cell functions. While a number of GO terms were found at day 14, none were significant at day 3 for either biological processes or cell components. The gene lists for all GO terms are provided as supplementary files.

## Discussion

The aim of the present study was to characterise alternative splicing events in response to 14 days of limb immobilization. The novel findings were that (1) numerous differentially alternative splicing events and differentially alternative spliced genes were evident throughout the 14 day limb immobilization period with a greater number of events at day 14 compared to day 3, (2) 37 GOBPs were differentially enriched after 14 days of immobilization including notable processes such as MAPK cascade, mitochondrion organization, and organelle organization, and (3) alternative splicing was more apparent in biological processes associated with the decrease of muscle protein synthesis (MPS) than increased muscle protein breakdown (MPB). There was a lack of significant GO enrichment terms at Day 3 despite having 699 DAS events. This may be due to inter-individual variability in the early time-course of immobilization, and/or an initial molecular response that lacked coordination before it stabilized into highly coordinated pathways after day 3.

The total number of alternative splicing events and alternatively spliced genes were consistently increased at day 3 and day 14 of immobilization compared with basal events and with a mean of > 10 transcripts per gene. This finding indicates an increased diversity of gene expression after skeletal muscle unloading may be due, at least in part, to increased splicing. Notably, SE was the most abundant form of AS events observed within our study of skeletal muscle from young healthy human participants. This is consistent with previous reports in different species, tissues, and physiological conditions, indicating that SE is the main splicing mode within global gene expression [16].

Sun and co-workers (2021) have previously identified an average of 2,000 differential alternative splicing events across 1,800 genes over a 7 day period using a rodent model of muscle unloading via hindlimb suspension. This corresponds to an average of around 1.1 AS events per gene, demonstrating widespread post-transcriptional regulation during muscle disuse in rats. Our knee brace immobilization study in humans revealed a lower number of differential alternative splicing events with 699 spliced events detected at day 3 and 1,004 spliced events at day 14. This difference highlights potential for variation in the magnitude and timing of alternative splicing responses between species and atrophy models, and modest differences in physiology and tissue-specific regulatory mechanisms. Nonetheless, each study supports the contention that increased AS responses in muscle to unloading are early and persistent over ~ 7–14 days [17, 18].

Whilst the total number of alternative splicing (AS) events remained relatively consistent between day 3 and day 14 in the present study, a closer examination revealed notable differences in the specific types of splicing events and the individual genes undergoing splicing changes. The increasing AS events and affected genes, due to the RI mechanism, between day 3 and day 14 suggests a time-dependent amplification of post-transcriptional regulation during prolonged muscle unloading, consistent with findings in rodent disuse [7]. Moreover, the specific genes undergoing splicing and the distribution of splicing modes differed between timepoints, reflecting a shift in regulatory processes as the effects of unloading progressed. This temporal divergence in splicing patterns indicates that longer durations of unloading are associated with increased complexity within transcriptomic regulation, which may lead to greater variability in protein isoform expression. Such molecular changes may underlie the progressive physiological and structural maladaptation observed with chronic skeletal muscle unloading [19].

Our study also indicates that the majority of the differential splicing events occurred within the coding sequence (CDS) region and may result in open reading frameshifts. This observation is consistent with what might be expected given the characteristics and coverage depth of the RNA-seq dataset used for the analysis. Other than changing the reading frame, frameshifted transcripts are also one of the conditions for activating the nonsense-mediated mRNA decay which would eventually degrade activated transcripts [20]. Whilst most alternative splicing (AS) events detected occurred within coding sequence regions, there were others identified in untranslated regions (UTRs), broadening the regulatory impact of splicing beyond protein-coding domains. The alternative splicing events at 3’UTR will either increase or decrease the binding site of miRNA and splicing that occurs in the 3’UTR may result in a decrease in length to avoid miRNA or RNA binding protein mediated regulation. It also greatly contributes to the proteome’s selectivity for translation and determines the outcome of mRNA [4]. Collectively, the synergistic actions of multiple mechanisms including AS likely contributed to the coordination of complex pathological processes in response to muscle unloading in the present study.

There were no GOBPs found to be significantly enriched for changes in AS at day 3, which may be due to a lower or variable magnitude of response for individual genes rather than a coordinated pathway response that was evident after 14 days. The pathways that were enriched at day 14 can be broadly categorised into three areas: remodelling pathways, developmental pathways, and mitochondrion-related pathways. Within remodelling pathways, specific processes such as intracellular protein transport and cellular localization were enriched, and these processes are associated with a decrease in amino acid transport capacity and protein signalling in response to muscle atrophy [21]. Our data indicates alternative splicing may downregulate amino acid transport proteins and contribute to the blunting of amino acid-stimulated anabolism with muscle unloading [21].

Just as mitochondria plays an important role in the muscle adaptation to exercise [22], the loss of mitochondrial homeostasis may be one of the primary instigators for the change in morphology and functional capacity of the muscle during unloading [23]. The present study identified AS associated downregulation of genes related to the inner and outer mitochondrial membrane cell components. A common outcome in experimental models of muscle atrophy is the reduction in mitochondrial volume which is evident from the downregulation of the mitochondrial membrane [24]. Indeed, we have previously undertaken proteomic analysis of muscle atrophy that also shows negative enrichment of the inner mitochondrial membrane organization and the outer membrane protein VDAC2 [8]. Agreement between AS events and proteome responses to unloading is supported by animal studies reporting a ~ 50% reduction of mitochondrial density in tibialis anterior muscles after two weeks of immobilization [25]. Therefore, AS may be an important part of the machinery associated with mitochondrial degradation during muscle atrophy, but further work will be required to clearly elucidate the contribution of AS in this regulatory process.

A focal point of skeletal muscle atrophy is the ubiquitin proteasome pathway [26]. However, in the present study none of the commonly identified ubiquitin ligases had significant differentially expressed mRNA with associated AS. We found that after 14 days there was downregulation of AS related to muscle protein degradation which may suggest AS may not be a primary mechanism in modulating MPB and instead may affect MPS in response to unloading. Intuitively, changes in MPS and MPB will occur in the maladaptive response that generates muscle atrophy but shifts in AS were more apparent in processes associated with the decline in MPS. However, while AS may not be directly involved as a mechanism in regulating the structural and contractile protein degradation process during atrophy, there are still potential AS contributions within signalling pathways controlling protein degradation.

The overarching analysis of this study has identified splice-altering events that may be promoting immobilization-induced skeletal muscle atrophy, with notable processes such as MAPK cascade, mitochondrion organization, and organelle organization affected. However, a limitation of this study is that individual differential splicing events were not confirmed by an independent experimental method. Accordingly, our findings are best interpreted as a characterization of global splicing dynamics during muscle unloading, with confirmation of specific events, and their translation to functional proteins, an important direction for future work.

## Conclusion

This is the first study to determine the extent of alternate splicing events and associated mRNA expression in response to unloading-induced atrophy in human skeletal muscle. The novel findings of the present study show the number of splicing events in muscle increases when habitual contractile activity is interrupted by limb immobilization, at least over the initial 14 days. Whether alternative splicing continues to advance as muscle unloading persists beyond 14 days remains to be established, but splicing is a regulatory step in gene expression to determine which part of the genetic sequence is finally used in synthesizing new proteins and can dictate protein variability in skeletal muscle. Accordingly, the novel characterisation of the biological processes and cellular components affected by alternative splicing in this study indicates splicing has an important role in the muscle cells response to unloading. Specifically, alternative splicing appears to promote down-regulation of target genes and subsequent differentially enriched biological pathways associated with the functional outcomes of decreased muscle mass and strength.

## Supplementary Information

Below is the link to the electronic supplementary material.

Supplementary Material 1 (XLSX 2.47 MB)

Supplementary Material 2 (CSV 275 KB)

Supplementary Material 3 (CSV 37.9 KB)

## Data Availability

All sequencing data that support the findings of this study have been deposited in the NCBI Sequence Read Archive (SRA) under the accession number PRJNA1462955. Data are publicly available and can be accessed via the SRA database.
